# Urban park system on public health: underlying driving mechanism and planning thinking

**DOI:** 10.3389/fpubh.2023.1193604

**Published:** 2023-06-12

**Authors:** Cheng Zhang, Qin Su, Yanqun Zhu

**Affiliations:** ^1^School of Geography and Tourism, Anhui Normal University, Wuhu, Anhui, China; ^2^Center of Urban and Rural Habitat Sustainable and Ecological Environment Planning Engineering Technology Research, Wanjiang University of Technology, Ma’anshan, Anhui, China

**Keywords:** city park, public health, ecology, driving mechanism, analytic hierarchy process

## Abstract

The driving mechanism and planning thinking of the impact of urban park system on public health the mission of urban geography, urban and rural planning or landscape architecture are to coordinate the relationship between people and places, people and nature. The municipal park system is an important part of the urban green space system. In order to effectively play the role of the urban park system in promoting the health of urban residents. This manuscript studies the coupling relationship between the “urban park system” and the “public health system” by building a coordination model, reveals the driving mechanism of the urban park system affecting the benign development of public health, and clarifies the positive driving effect of urban parks on public health. Finally, based on the analysis results, the manuscript considers the optimal development strategy of urban parks from the macro and micro levels to promote the sustainable development of urban public health.

## 1. Introduction

The process of urbanization is still advance. The sharp increase of urban population has resulted in the decline of environmental quality and the threat of ecological stability. The negative impact of urban space environment on public health is gradually exposed (1). On 25 October 2016, the Central Committee of the Communist Party of China and the State Council issued the Outline of the “Healthy China 2030” Plan, which deployed plans to enhance public health from the policy level. The fifth part of the outline highlighted the importance of “building a healthy environment,” clarified its construction rules, and advocated strengthening the governance of environmental issues affecting health and promoting the healthy development of the people (2, 3). As a key component of urban green space system, urban park green space has a significant effect in purifying urban air and beautifying public environment.

At present, scholars at home and abroad have made many academic researches on the role of urban park system in promoting the healthy development of residents: Jiang Bin, Zhang Tian, William C. Sullivan and others discussed the impact mechanism of urban green landscape on public health (4), and explained the impact of urban green landscape on public health and well-being through five theoretical mechanisms. Yao Yanan and Li Shuhua reviewed the research on urban green space based on public health (5) and concluded that the mechanism of green space positively affecting public health may be to provide ecological products and services through natural elements in green space and promote healthy behaviors. Maming, Bob Mogol and others studied the factors that affect physical activity in green open space design (6), combed six factors that affect physical activity based on space, place and perception, and established a preliminary indicator framework for physical activity factors in green open space. Green sport can bring positive short-term and long-term health results. Barton studied the best plan of nature and green sport to improve mental health (7), analyzed and evaluated the best plan of green sport needed to optimize self-esteem and emotion.

The research perspective on the impact of urban parks and green spaces on public health in European and American countries is unique and innovative: Zhao Xiaolong, Wang Mincong, and others studied the interaction between the planning and design of urban parks in Britain and the evolution of public health themes from the perspective of public health and well-being (8). In combination with social, political and economic background, health conditions and other factors, the public health appeal is described in six stages: preventing infectious diseases, solving urban environmental problems, paying attention to leisure sports, seeking social integration and promoting mental health, cultivating a healthy lifestyle, and building a common health of society and ecosystem.

Zhang Mengjia and others analyzed the classification system of urban parks in the United States (9). The urban park system of the United States has significant functions in promoting physical activity of its residents and improving the level of national public health. Therefore, one of the characteristics of its urban park classification is “physical activity demand oriented.” The study takes Minneapolis city park system as an example to conduct in-depth research, and draws important experience and suggestions on China’s “building a healthy China”: re-examine the functional positioning of domestic city parks. City parks have become an important breakthrough in optimizing national fitness venues, and actively establish a classification system of city parks that is suitable for national conditions and oriented by physical activity needs, At the same time, pay attention to the “accessibility” indicators of urban parks.

A large number of studies above comprehensively revealed the mechanism of urban park system on public physical and mental health from multiple perspectives, but ignored the overall driving role of public “social health.” Although some studies included “improving social capital” into the utility scope of urban parks, they did not consider social functions such as “improving esthetics and mitigating natural disasters,” which is one-sided. This paper explores the social health effects produced by the urban park system in combination with relevant domestic and foreign studies, uses the principal component analysis method and the analytic hierarchy process (AHP) to build an evaluation index system of the ability of urban parks to promote public health development, constructs a coordinated development model to clarify the coupling relationship between the urban park system and public health, and reveals the driving mechanism of the urban park system to drive public health development, Clarify the detailed relationship between “promoting physical activity, creating ecological value, improving viewing experience, and enhancing social viscosity” and the driving effect of public health, to explain how the urban park system promotes the positive development of public health.

## 2. Methods

### 2.1. Influencing factors of human health

**Figure 1:**
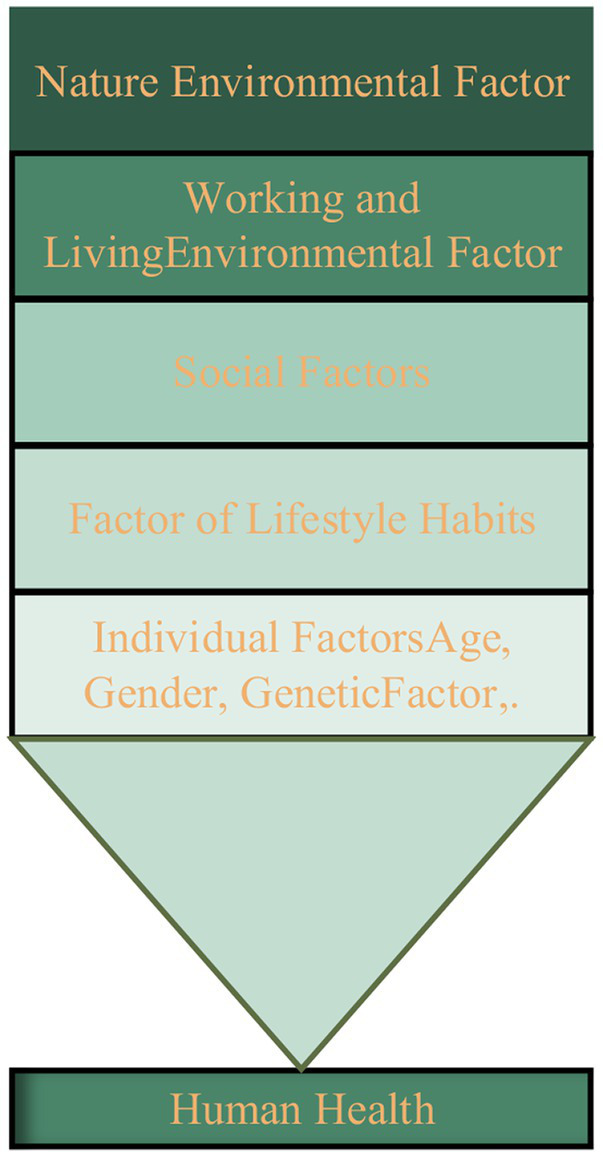
Distribution map of factors affecting human health.

Human health is influenced by the combined effects of dominant and recessive factors, and the order and level of each factor’s impact are different. According to the relevant research background (4), the distribution map of factors affecting human health is drawn, as shown in Figure 1.

Individual factors, factors of lifestyle habits, social factors, working and living environmental factors, and natural environmental factors seriously affect human health. While factors at various levels directly affect human health, there may be interactions: for example, the choice of human life and work style is due to social factors, the consideration of living and working factors; the way, field and space of public social interaction depends on the urban environmental conditions; the interaction of multiple factors gradually changes the human health status, and fundamentally reflects the level of social public health. The factor distribution map shows that the root cause of human health changes lies in external environmental factors. Urban parks are an important component of the external environment in which modern human beings live. Therefore, urban parks have an comprehensive irreplaceable effect on human public health, even exceeding the impact of human factors on health.

### 2.2. Principal component analysis

#### 2.2.1. Determination of principal components

The principal component analysis method brings multidimensional factors into the same system for quantitative research, and describes the performance of the system with fewer evaluation indicators by simplifying complex impact indicators. The theory is relatively perfect and the statistical performance is relatively diversified. Based on the principal component analysis method, the main evaluation index of the evaluation index system, namely the content of the criteria layer, is determined to reduce the calculation amount of the evaluation of the ability of urban parks to promote public health development. Standardize the evaluation index data, and calculate the index. The correlation coefficient matrix is defined as R. The principal component calculation is applied to the three key variables of matrix R, namely, eigenvalue, variance contribution rate and cumulative contribution rate, which are marked as λi, Bi, Mm, the calculation method of variance contribution rate and cumulative contribution rate is shown in formulas (1, 2):


(1) $$D_{i}=\frac{\varepsilon_{i}}{\overset{p}{\underset{i=1}{\sum }}\varepsilon_{i}}$$

(2) $$H_{m}=\overset{m}{\underset{i=1}{\sum }}D_{i}$$

In the formula, the information amount of each principal component is described by D_i_, and the information synthesis ability of the first m principal components is described by H_m_. Take the first level indicators evaluating the ability of urban parks to promote public health development as the principal components, calculate their eigenvalue, variance contribution rate and cumulative contribution rate, and further determine the simplified principal components that can express the ability evaluation of urban parks.

#### 2.2.2. Weighted comprehensive grade calculation

The weighted summation method is used to calculate the comprehensive score of the evaluation on the ability of urban parks to promote public health development. The calculation formula is shown in formula (3):


(3) $$J=\omega_{1}\times G_{1}+\omega_{2}\times G_{2}+\cdots +\omega_{i}\times G_{i}$$

Among them, the indicator weight is ω_i_ represents, G_i_ represents the principal component, and its calculation formula is:


(4) $$G_{i}=\overset{l}{\underset{x=1}{\sum }}q_{xi}\times A_{nx}$$

Among them, the factor load coefficient of the x index of the i principal component is expressed in q_xi_, and the standardized form of the x evaluation index is expressed in A_nx_.

### 2.3. Coordinated development model

Whether multiple systems are coordinated and properly coordinated can be reflected through “coupling degree” to judge whether they form a virtuous circle, clearly express the strength and interaction degree of the system or elements, and master the process of interaction between systems. The relationship between systems is complex and unbalanced, and the level of collaboration is good or bad. Therefore, the concept of “coordinated development degree” is used to evaluate the level of coordinated development between systems and the degree of both, so as to more accurately express the overall coordination level and synergy of the system. The coordinated development degree model consists of coupling degree model and coordinated progress degree model. Urban parks generate supply and public groups generate demand, so there is a definite coupling relationship between the two. In order to more accurately describe the driving impact of the urban park system on public health, this study uses the coupling degree to express the driving relationship between the two. When the coupling degree is advantageous, it is considered that the driving effect is good.

## 3. Empirical research

### 3.1. Overview of the study area

**Figure 2:**
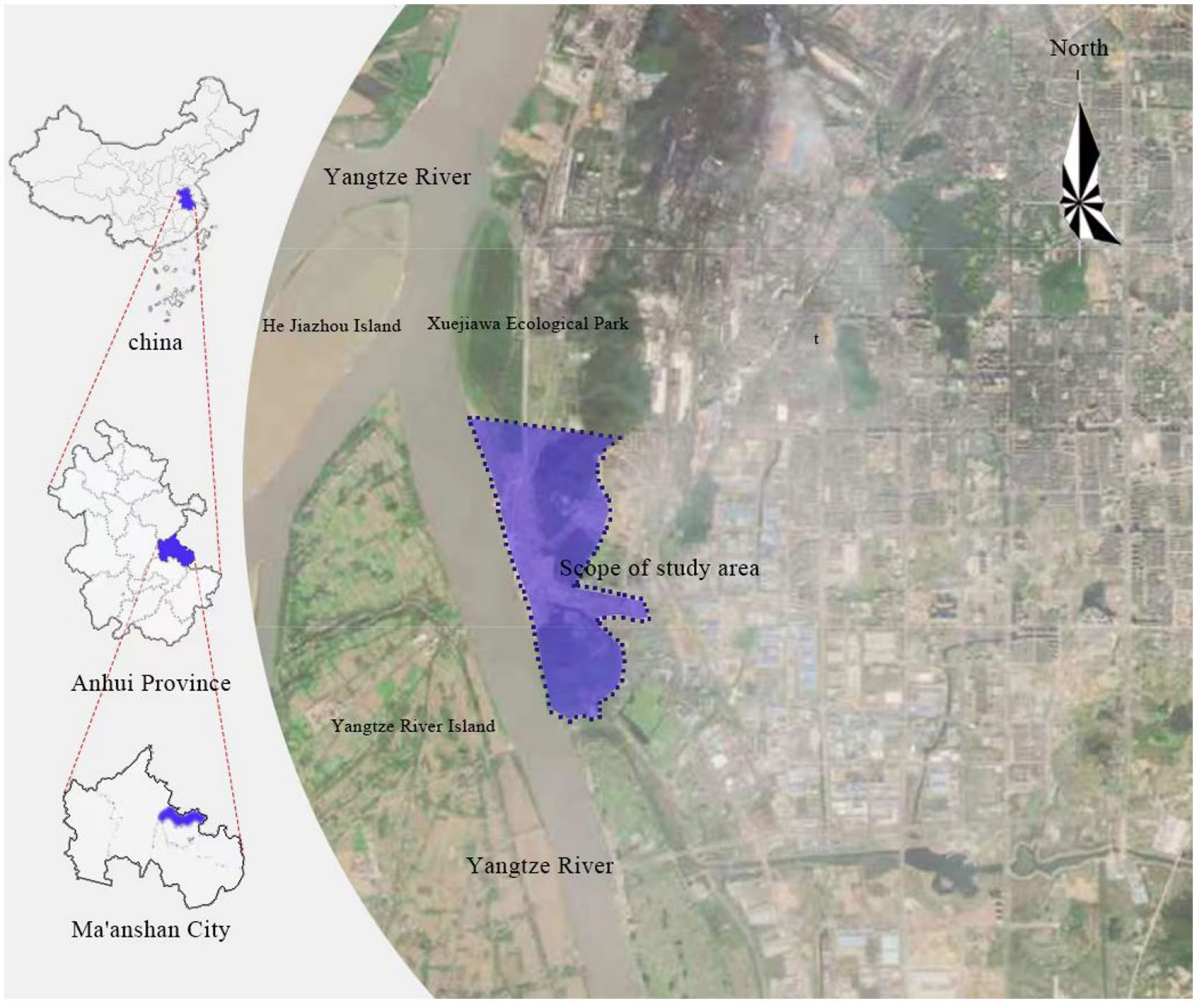
Overview of the Study Area.

The Caishiji Riverside Cultural Park in Ma’anshan, Anhui Province, China is chosen as the research object. The research area is situated in the north of the Yushan District Quarry Scenic Area in Ma’anshan City. It is the only living shoreline of the city near the river in terms of ecological protection, extending the original quarry scenic spot in area, and creating a riverside ecological and cultural place for the public to live by virtue of its profound historical and cultural heritage. Caishiji Riverside Cultural Park is adjacent to the Yangtze River in the west, Liufen River in the north, Xishan Mountain, Xiaojiuhua Mountain and Hebao Mountain in the east, and Suoxi River in the south. It covers an area of 3.54 square kilometers, as shown in Figure 2. The park has been planned and laid out with “three parks and two axes” as the core. “three parks” refers to the three regional settings in the north, south and middle of the park: (1) it shows the development process of Ma’anshan’s industrial civilization and keeps pace with the era of ecological and environmental protection. (2) The south area of the park is close to Caishiji, where there are a series of cultural parks, such as the Buddhist Culture Park of Xiaojiuhua Mountain, the War Relics Park of Hebao Mountain, and contains a long and touching historical and cultural connotation. (3) The central area of the park is designed as a leisure park, because the terrain of this part is relatively flat, with a safe natural environment, and a supporting leisure complex has been built. The “two axis” refers to the “north–south landscape ecological axis” formed by the original wetland and the “east–west development axis” formed by the connection with the urban center. The intersection of the two axes is built into a square park.

For evaluating the ability of urban parks to promote public health development in Caishiji Riverside Cultural Park, so as to build a driving mechanism for the impact of an urban park system on public health, and obtain relevant thinking on urban park planning. This park is under a large flow of people, rich water landscape and green space, and complete cultural facilities. It is a great place for local residents to provide daily entertainment, fitness, and social interaction. A questionnaire survey was undertaken among 50 residents within the park. Each resident distributed two questionnaires. One questionnaire investigated the residents’ evaluation of the park’s value creation, and the other questionnaire investigated the relevant issues of public health, including “physical health, mental health, and societal health.” The above questionnaires were used as data samples for the evaluation of the park’s ability to drive public health. In addition, in-depth interviews were conducted with residents on pertinent issues. Fifty respondents, composed of men and women of all ages, have certain research value and reliability. The detailed information of the respondents is given in Table 1.

**Table 1:** Detailed information statistics of questionnaire respondents.

Research elements	Type and number of people					
Gender	Male (24 persons)			Female (26 persons)		
Age	Under 14 years old (2 persons)	15–18 years old (4 persons)	19–25 years old (5 persons)	26–40 years old (15 persons)	41–65 years old (12 persons)	Over 65 years old (12 persons)
Education	Junior high school education and below (4 persons)	High school education (12 persons)	Higher vocational college degree (22 persons)	Bachelor degree (8 persons)	Master degree (3 persons)	Doctoral degree (1 person)
Park activity stay time (hours)	0.5 h and below (10 persons)	0.5–1 h (12 persons)	1–2 h (10 persons)	2–4 h (14 persons)	4–6 h (2 persons)	More than 6 h (2 persons)
Purpose of park activities	Fitness and exercise (25 persons)	Accompany the older adult and children (24 persons)	Social networking with friends (12 people)	Collective activities (7 persons)	Walking and leisure (24 persons)	Cultural exchange (8 persons)
Park activity period	Before 7:30 a.m. (10 persons)	7:30 a.m.–11:30 a.m. (14 persons)	11:30 a.m.–2:00 p.m. (2 persons)	2:00 p.m.–5:00 p.m. (16 persons)	5:00 p.m.–7:00 p.m. (6 persons)	After 7 p.m. (2 persons)

### 3.2. Determination of principal components of evaluation indicators

**Figure 3:**
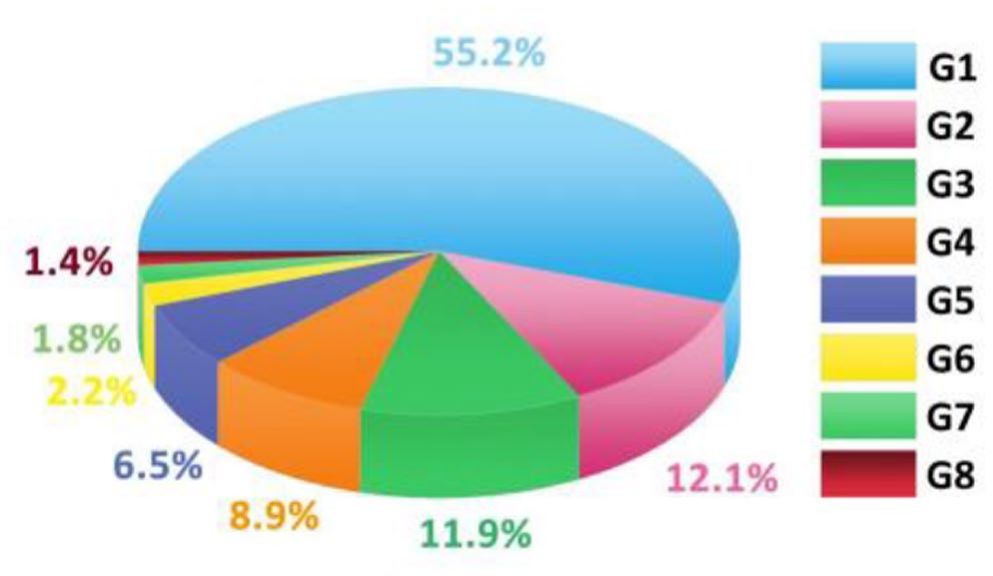
Statistics of contribution rate.

The expert team preliminary determined that there are 8 primary indicators for the evaluation of the ability of urban parks to promote public health development, which are respectively: promoting physical activity (G1), promoting cultural communication (G2), creating ecological value (G3), improving viewing experience (G4), creating entertainment atmosphere (G5), enhancing social viscosity (G6), creating leisure experience (G7), and enhancing esthetic meaning (G8). Based on formulas (1, 2), the contribution rates of the eight principal components are calculated, sorted from high to low, and their cumulative contribution rates are calculated, as shown in Figure 3.

The factors with a cumulative contribution rate of more than 85% are taken as the principal components. According to the analysis of the data in Table 2, the cumulative contribution rate of the first four factors G1~G4 is 88.1%, which meets the standard that the cumulative contribution rate is more than 85%. Therefore, the first four factors “promote physical activity, create ecological value, improve viewing experience, and enhance social cohesion” are taken as the principal components of the evaluation of the ability of urban parks to promote public health development, which is the final criterion layer. It achieves the purpose of expressing the evaluation of park capacity and reduces the amount of calculation.

**Table 2:** Comparison of relative importance of criteria level indicators.

Criterion level indicators	Promote physical activity (B1)	Create ecological value (B2)	Improve viewing experience (B3)	Enhance social viscosity (B4)
Promote physical activity (B1)	1	2	3	5
Create ecological value (B2)	1/2	1	2	3
Improve viewing experience (B3)	1/3	1/2	1	2
Enhance social viscosity (B4)	1/5	1/3	1/2	1

### 3.3. Construction of evaluation index system and calculation of index weight for urban parks’ ability to promote public health development

**Figure 4:**
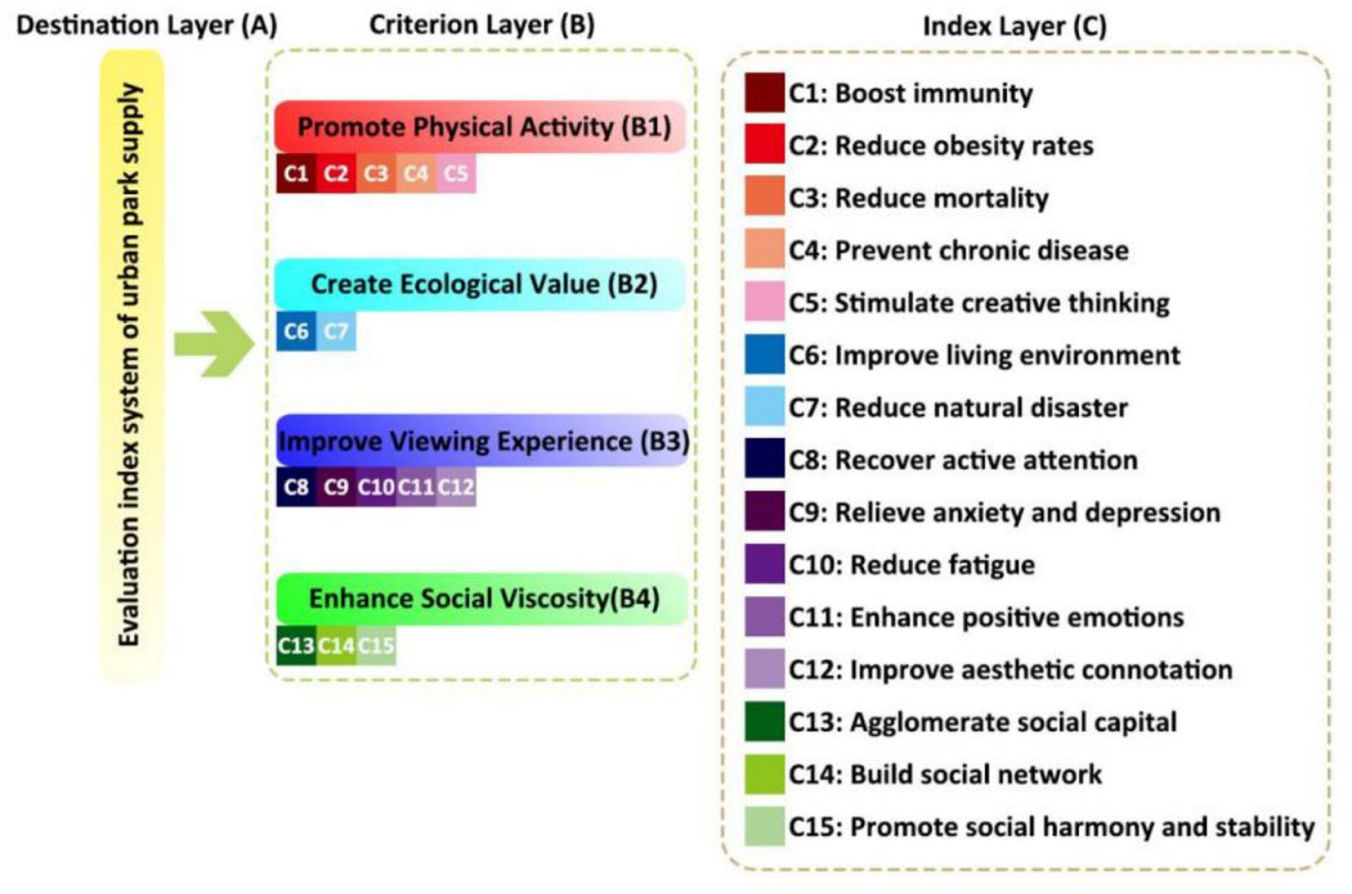
Evaluation index system of urban park supply. **(A)** destination layer; **(B)** criterion layer; **(C)** index layer.

According to the results of the principal component analysis, it is determined that the main factors for urban parks to promote the development of public health include promoting physical activity, creating ecological value, improving viewing experience, and enhancing social cohesion. As primary indicators, each primary indicator includes a total of 15 secondary indicators. The construction of the urban park supply evaluation indicator system is shown in Figure 4.

In this study, 20 experts were given two factor comparison questionnaires, and different matrix construction results and different weight calculation results were obtained based on 1–9 scale method; Then calculate the arithmetic mean value of the weight results obtained by 20 experts in the field as the final index weight results. Table 2 shows the judgment matrix of the criteria level of the evaluation index system for the ability of urban parks to promote public health development.

The figures in Table 2 indicate the relative importance of the two evaluation indicators for the target layer. For example, under the goal of promoting public health in urban parks, the importance of “improving viewing experience” is 1/2 compared with “creating ecological value.”

It is calculated that the weight of indicators at the criterion level is to promote physical activity—3.107, create ecological value −1.442, improve viewing experience −0.693, and enhance social viscosity −0.322, with normalized weights of 0.534, 0.208, 0.144, and 0.114, respectively. Finally, the indicator weight passed the consistency test, and the calculated weight results can be used as valid data (10).

**Figure 5:**
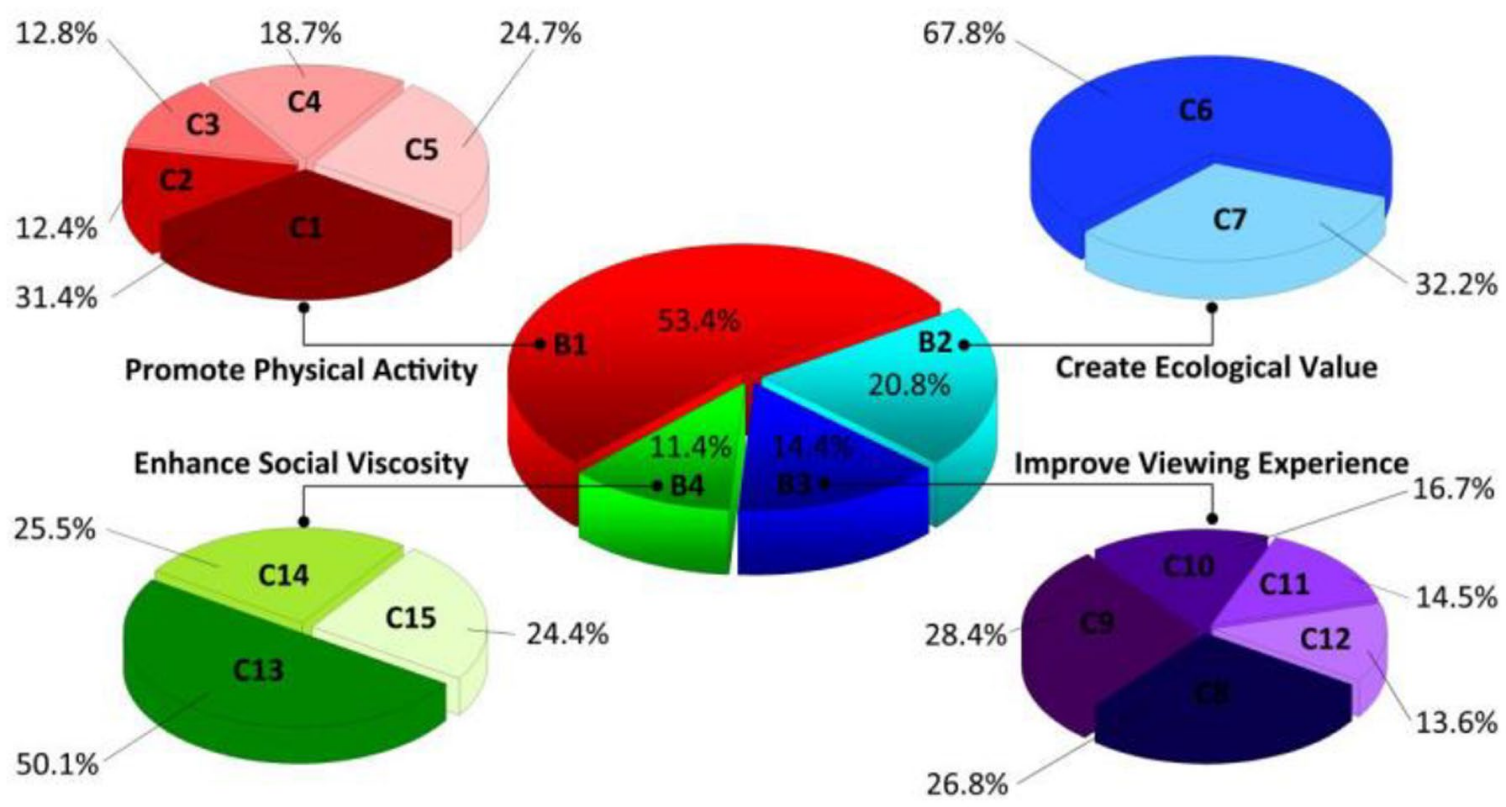
Weight of urban park supply evaluation index.

**Figure 6:**
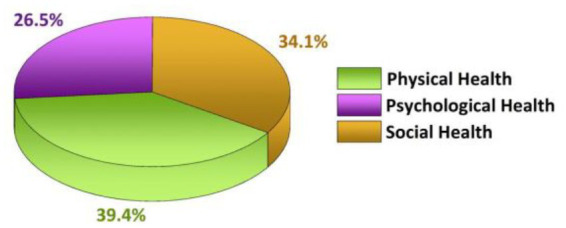
Weight of public health evaluation indicators.

According to the above calculation method and consulting experts’ opinions, the survey results were integrated with reference to relevant literature, a judgment matrix was constructed, and finally the weights of urban park supply evaluation indicators and public health evaluation indicators were calculated as shown in Figures 5, 6.

Using the formula (3) weighted summation method, it is calculated that the park driven public health capacity in the study area is classified into three levels, and the evaluation level is divided into five levels. The level of the park driven public health capacity in the study area is medium. The “target layer” realizes the improvement of public health in the “indicator layer” through the “criteria layer.” Therefore, in this logical relationship, the four factors of “promoting physical activity, creating ecological value, improving viewing experience, and enhancing social cohesion” are coupled with the “physical health, psychological health, and social health” of public health. The former plays a driving role in the latter, and becomes the factor of “driving” the development of public health in urban parks.

### 3.4. Construction of coordinated development degree model

This study uses coupling degree to express the driving relationship between the two to objectively interpret the driving impact of urban park system on public health. When the coupling degree is good, it is considered that the driving effect is good. Based on the accuracy and feasibility of data acquisition, from the perspective of the value created by urban parks and the benefits obtained by public residents, two subsystems of “urban park supply” and “public health demand” are constructed. The “urban park supply” subsystem contains four positive indicators, and the “public health demand” subsystem contains three positive indicators. Based on the analysis results in Table 3, “promote physical activity, create ecological value, improve viewing experience, and enhance social viscosity” is defined as the supply indicator of urban parks, and “physical health, mental health, and social health” is defined as the demand indicator of public health.

**Table 3:** Grading of coordinated development stage (driving stage) of subsystems.

Coordinated development	0~0.39	0.40~0.49	0.50~0.59	0.60~0.69	0.70~0.79	0.80~0.89	0.90~1.0
Coordination level division	Maladjustment stage	Approaching maladjustment	Basic coordination	Primary coordination	Intermediate coordination	Good coordination	Very coordinated

Calculate the fuzzy membership function of the obtained urban park index weight, and obtain the membership value of the urban park supply index; The comprehensive evaluation index of urban park supply Xi and the comprehensive evaluation index of public health demand Yi are obtained based on the weighted average method. The specific methods are shown in Formulas (5, 6):


(5) $$X_{i}=\overset{4}{\underset{i=1}{\sum }}\;\;\;\;\;\;\;\;\;x_{j}\times a_{j}$$

(6) $$Y_{i}=\overset{3}{\underset{i=1}{\sum }}\;\;\;\;\;\;\;\;\;y_{j}\times b_{j}$$

Where, x_j_ represents the membership value of park supply indicator j, and y_j_ represents the membership value of public demand indicator j; Entropy weighting coefficient is described by a and b. Based on this, the coordinated development model of urban parks and public health is constructed as follows:


(7) $$P=\sqrt{C\times T}$$

(8) $$\lambda ={\left[\frac{X_{i}\times Y_{i}}{{\left(\frac{X_{i}+Y_{i}}{2}\right)}^{2}}\right]}^{r}$$

(9) $$G=\alpha \times X_{i}+\beta \times Y_{i}$$

In the formula, the coordinated development and coupling degree of urban park supply and demand are, respectively, P, λ. The comprehensive evaluation index is expressed as G; The adjustment coefficient and undetermined coefficient use r and α, β. It means that the former value is 2 and the latter value is 0.5, respectively.

The relationship between urban park supply subsystem and public health subsystem is defined by reference to the ArcGIS natural breakpoint classification method using numbers: (1) Antagonism stage: 0 < coupling degree ≤ 0.4; (2) Running in stage: 0.4 < coupling degree ≤ 0.8; (3) Coordination stage: 0.8 < coupling degree ≤ 1. The coordinated development stage (driving stage) of the two subsystems is further classified, and the results are shown in Table 3.

## 4. Driving mechanism and planning thinking

### 4.1. Driving mechanism

Urban parks are external environmental factors that affect human health and significantly affect public health (11). It can be seen from the comprehensive evaluation results of public health level driven by parks in the study area that among the four public health drivers, public groups can have a positive reaction of “promoting physical activity” when visiting urban parks. The weight value of 0.534 proves that “promoting physical activity” is a significant function of urban parks to drive the development of public health, and it brings health benefits for the public at the physiological and psychological levels; The weight value of providing ecological services is 0.208, which is second only to “promoting physical activity” in driving the development of public health; The weight of providing ornamental services is 0.144, which has played a positive role in driving the development of public health; Although the weight of social capital is only 0.114, it plays a positive role in improving capital accumulation, increasing positive emotions and social harmony and stability.

**Figure 7:**
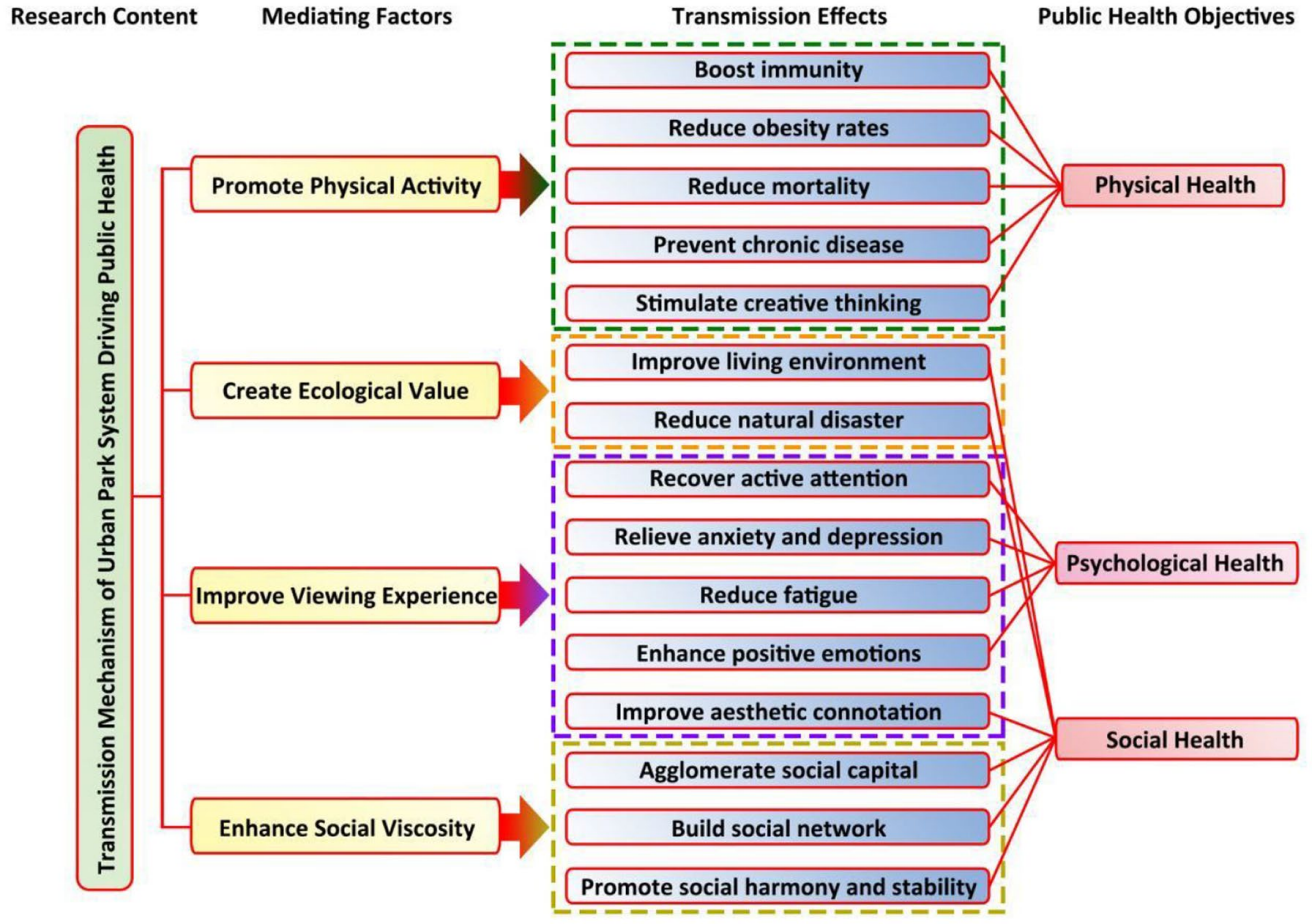
Driving mechanism.

Based on the coordination degree model, the coupling relationship between the urban park supply subsystem and the public health demand subsystem is calculated. The level of their coordinated development is “good coordination,” and the coordinated development degree is 0.81. Moreover, the comprehensive evaluation index of the urban park supply is greater than the comprehensive evaluation index of the public health demand, presenting a health impact situation of supply exceeding demand. It can be seen that the urban park system has a positive impact on public health, forming a positive driving mechanism. According to the research results, the mechanism of urban park system driving public health is shown in Figure 7.

It can be seen from the analysis of Figure 7 that the driving function of urban parks for public health is mainly manifested in three levels: “physical, psychological and social.” The process of driving public health by urban parks includes four intermediary factors: “promoting physical activity,” “creating ecological value,” “improving viewing experience,” and “enhancing social viscosity,” which further generate a positive public health driving effect and ultimately achieve the goal of public physical health, psychological health, and social health.

### 4.2. Driving mechanism analysis

#### 4.2.1. Driving mechanism of urban parks for public “physical health”

1. Enhance immunity and reduce obesity rate. Urban life has led to great pressure on public life and work, and the frequency of physical exercise and physical activity has decreased. Urban parks have become an important link for the public to increase the number of physical activities (12). A large number of practical studies have proved that the closer the living area is to the city park, the stronger the willingness of physical activity is. Residents can generate physical activity through the city park to enhance the immunity of the urban population and reduce the obesity rate.
2. Reduce mortality. Walking in urban parks with dense trees and vegetation is beneficial to increase the scale of adult pain resistant active protein, avoid accelerated secretion of adrenaline, curb the incidence rate of related diseases, prolong the life span of the older adult population, and reduce mortality; Pregnant women living near urban parks have a high frequency of activities around green vegetation, and the probability of premature delivery and underweight infants is relatively low.
3. Prevent chronic diseases. Inhaling polluted air can easily lead to respiratory diseases and other chronic diseases. Garden plants can release oxygen, absorb pollutants, inhibit bacteria, and improve the quality of living air (13–15).
4. Stimulate creative thinking. Physical activity and exercise can easily lead to positive emotions. Proper physical activity in the park system can help increase individual’s sense of energy and reduce tension. When people are dominated by positive emotions, they do not need to use more psychological resources to solve problems, but focus on creative thinking, which is more likely to show highly creative behavior, thus creating a good atmosphere for creative thinking. Therefore, the urban park system creates a place for individual activities and indirectly stimulates individual creative thinking.

The following is the interview content of park residents sorted out in this study (the first female resident and the first male resident are expressed by F1 and M1 respectively, and so on):

Author: Do you often go to Riverside Culture Park for physical activities, and do you have any changes in your physical feelings, health status, and thinking state? You can talk about your life experience in terms of weight change, immunity, creative thinking, etc.

Resident (F1): I basically come here every morning and afternoon for activities. The air here is very good, and the air humidity in the adjacent waters is very good and comfortable; I feel comfortable when I come to the park for activities. I feel much less physical burden. Recently, the chance of catching a cold has obviously decreased due to the severe flu.

Resident (F2):Every time I am upset by my work, I will come here to relax and do some exercises. My mood will be relaxed and I will have more desire and confidence to solve the problems of work.

It is obvious from the residents’ answers that their physical condition and thinking state have been significantly improved through physical activities in Riverside Culture Park, which has promoted the residents’ physiological health.

#### 4.2.2. Public “psychological health”

1. Active attention recovery. The green natural environment is a typical “restorative environment.” Human exposure to the green natural environment can produce “stress recovery response,” reduce tension and restore attention.
2. Relieve anxiety and depression, and release psychological pressure. The ornamental function of urban parks is more prominent, creating a space environment for relaxation, meditation and appreciation of plant water bodies (16).
3. Reduce fatigue. Modern urban park design is generally based on a large amount of research on the public’s esthetic taste and practical needs. The planning and design of the park system is remarkably systematic, realizing the organic combination of individual “psychology - spirit - external environment.” Therefore, urban parks are not only leisure places to reduce physical fatigue, but also outdoor spaces to remove psychological fatigue. It was mentioned in the interview that:

Visitor: What time do you choose to come to the park for leisure activities? What changes do leisure time make you?

Resident (M1): On weekends and holidays, they usually come with their families. They usually have less rest time and have a lot of work pressure. Playing in the park allows me to relax my tense nerves. My depression and anxiety at work are reduced. Seeing the green landscape and cultural influence, the whole person is relaxed.

Resident (M2): I usually have a lot of study pressure. I live near here. I often come here with my friends during holidays to see the green trees to relieve my visual fatigue; When I came home from the park, I felt very comfortable, and my idea of doing homework became broader.

According to the interview with residents, the physical activities of residents in Binjiang Park can reduce their psychological fatigue, relieve their anxiety, and release their psychological pressure, thus realizing their psychological health.

4. Increase positive emotions. In the field of epidemiology in the United Kingdom, the impact of environmental beauty on public health has been studied in depth. With a large number of beautiful landscape images as data samples and multiple regression analysis as a tool, it has been confirmed that both landscape beauty and greening are conducive to public health. The pleasant scenery of urban parks gives people visual enjoyment, gradually eliminating the public’s sense of loss and negative emotions, and increasing positive emotions (17, 18).

#### 4.2.3. Public “social health”

1. Improve the living environment. In the 1960s, American epidemiology had found the law of high mortality rate of urban high temperature heat wave. For example, 85% of the more than 200 deaths in St. Louis in July 1966 occurred in cities. It can be seen that the urban heat island effect leads to local climate change, which increases the mortality of urban population. Under the effect of urban heat island, the intensity of high temperature in urban areas is intensified. The temperature in urban areas is slow at night. Urban residents are under the heat stress of alternating black and white for a long time. The mortality of people with poor physical conditions is greatly increased. In addition, respiratory diseases caused by urban air pollution have become a great threat to human survival. The ecological value created by the urban park system plays a positive role in air purification and greenhouse effect mitigation, and cannot be replaced in improving the living environment (19).
2. Mitigate natural disasters. In recent years, extreme weather, climate anomalies and other factors often lead to flood disasters. In addition, the construction of urban drainage systems is very different, and the construction of sponge cities is insufficient. The surface water infiltration of many urban streets is difficult, which increases the probability of flood disasters. The urban park system includes large-scale forest vegetation, which has played a positive role in blocking floods and pollutants.
3. Improve the esthetic connotation. In recent years, the planning and construction of China’s urban park system has favored “cultural inheritance and development,” and the layout and artistic expression of urban parks have been carried out by drawing on the techniques of creating beauty from Chinese classical gardens, which not only shows the unique esthetic connotation, but also is of great benefit to the pleasure of individual material life and spiritual life. Urban residents unconsciously accepted the influence of garden art when they were enjoying the park. The beauty of artistic conception and form displayed in the park design has imperceptibly improved the individual esthetic connotation and taste.
4. Gather social capital. Social capital generally refers to the characteristics and resources of social organizations brought about by the association between individuals or groups to optimize social efficiency. Although the weight of social capital is only 0.114, it plays a positive role in improving capital accumulation, increasing positive emotions and social harmony and stability. A large number of studies have confirmed that strong social ties benefit from the support and maintenance of urban park green space, outdoor gathering activities and other factors. Urban parks create frequent social opportunities, strengthen social ties between public groups, and maintain existing social capital; The groups that go to the common city park become familiar gradually, driving the public to carry out social contact activities regularly and repeatedly, forming an expanding social bond beyond the scope of daily social contact, and taking common interests as the carrier to improve the degree of capital accumulation.
5. Build social networks. In the information age, the Internet has become the main virtual tool for modern people to socialize. The urban park system has become a diversified real space for residents to relax and socialize. It has derived the static social models of tree lined paths, natural landscapes, sunshine lawns, water games, sports and other dynamic social models, as well as the organizational social models of fitness organizations, dating organizations, environmental protection organizations, which constitute the link between urban residents, It is helpful for urban residents to build and expand their own social networks. The construction of the urban park system has avoided the closed communication defects brought by the virtual social way, enhanced the social viscosity, and promoted the healthy development of public social communication. It was mentioned in the interview that:

Interviewer: Do you usually come here for activities or do some leisure projects with others?

Resident (F3): I came here to chat with friends. The ecological environment here is good, with trees, water and cultural landscape. I can find many topics when chatting with friends.

Resident (M3): Some retired friends and I often get together here. There is enough time in the daytime to talk about poetry and historical stories. The historical and cultural atmosphere here is particularly good.

It can be seen from the interview that Riverside Culture Park creates a good outdoor space for group activities, provides a private and eco-friendly place for friends to socialize and cultural exchanges, and helps residents achieve the goal of social health.

6. The society is harmonious and stable. The foreign research on the relationship between tree canopy and crime rate shows that increasing 10% canopy coverage can reduce the crime rate by about 12%; There was a study in Chicago that found that most of the living places of groups with low crime rate and stress behavior frequency were green vegetation spaces, and the safety level of such areas was high; Therefore, good park vegetation coverage can strengthen social equality, effectively enhance social cohesion, reduce criminal acts, and contribute to social harmony, security and stability.

### 4.3. Planning for urban park system construction

**Figure 8:**
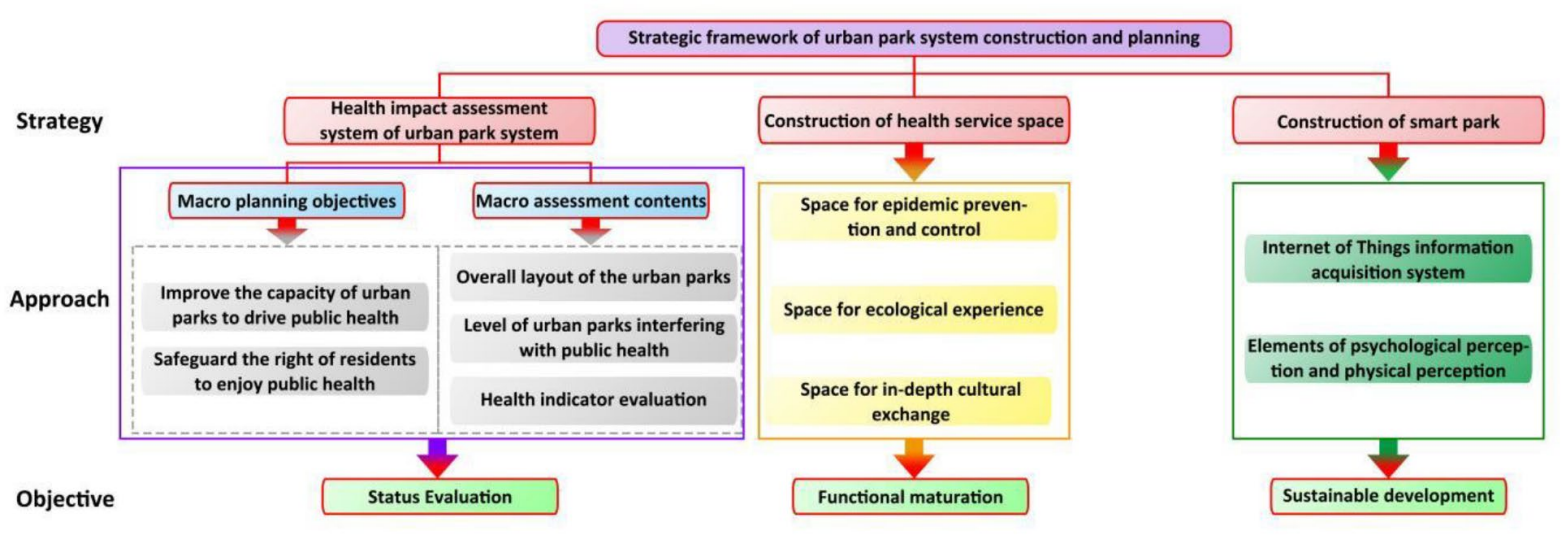
Thinking framework of urban park system construction planning.

The driving mechanism of the impact of the urban park system on public health built in this study not only considers the psychological and physical health aspects, but also integrates into the social health goals. The analysis of the public health impact mechanism is relatively comprehensive, but the evaluation system of the impact of the urban park system on public health is not perfect and lacks detailed rules for evaluation; At present, the urgent situation of the COVID-19 epidemic urgently needs to build an outdoor epidemic prevention and control space to enhance the ability of social epidemic prevention; The application of artificial intelligence technology in park data collection and environment perception is increasingly mature, which can quickly and timely capture the dynamics of the park environment and reduce the cost of manual management (20–24). Based on the above considerations, a framework layout has been made for the construction of the urban park system, as shown in Figure 8.

#### 4.3.1. Construction of urban park system health impact assessment system

Only by correctly recognizing the existing problems and advantages of urban parks can we formulate reasonable planning strategies in a timely manner. Therefore, it is urgent to build a health impact assessment system for the urban park system to clarify the direction of improvement of the urban park system. The health impact assessment technology in the field of green space planning in developed countries in Europe and the United States has developed relatively well, forming a systematic theoretical system. As the theoretical and practical support for the healthy development of cities, the health impact assessment technology has been applied to the green space planning projects of urban parks. The study preliminary constructs the health impact assessment system of urban parks, as shown in Table 4.

**Table 4:** Health impact assessment system of urban park.

Planning level	Macro planning	Intervention program	Micro-planning
Assessment target	Optimize the ability of urban parks to optimize public health, and ensure that urban residents enjoy equal rights to public health.	Optimizing the degree of closeness of development control to public health.	Pay attention to the microscopic scenery of urban parks and strengthen the control of monitoring functions.
Evaluation content	The overall structural layout of urban parks, health indicators, and the level of indicators that interfere with public health.	The structural layout of plants, water bodies and hard buildings, the indicators of the elements of the garden, and the level of indicators that interfere with public health.	The layout and structure of micro-elements, the level of micro-elements interfering with public health.

Table 4 outlines the objectives and contents of the three stages of “macro planning,” “intervention planning,” and “micro planning,” providing reference for accelerating the construction of the health impact assessment system of urban parks. The health impact assessment technology of urban parks adheres to the principle of “health promotion” (25, 26). Considering the impact way and extent of the green space planning results of urban parks on public health, the planning decision-makers can adjust the landscape design scheme according to the health impact assessment report to optimize the public health function of urban parks. In addition, the health impact assessment has identified ways for urban park planning to improve health, and has used limited capital investment to play a greater ecological and social benefits. China urgently needs to speed up the construction of the urban park green space health impact assessment system, enhance the public health awareness of decision-makers, achieve regional economic and cultural prosperity by optimizing public health, and promote the sustainable and healthy development of the urban park system.

#### 4.3.2. Add health service space

The construction of health service space focuses on epidemic prevention and control space, and the severe epidemic situation highlights the key of emergency sites. With convenient transportation, open space and good ecological environment, urban parks are the best choice for epidemic prevention and evacuation, disaster prevention and mitigation. The central area of Caishiji Riverside Culture Park is a leisure park, with flat terrain and good greening, which is suitable for use as a flexible space for epidemic prevention and emergency services to meet the diversified needs of emergency events (27, 28).

#### 4.3.3. Smart park layout

The smart park layout applies diversified intelligent management technologies to coordinate the integrated development of environmental, social and economic benefits. Spatial pattern design, plant population planning, and public facilities building, these basic park planning and management data can be recorded in the form of the Internet of Things, which combines the Internet of People and the Internet of Environment. The information feedback and update system of Smart Park provides data support for future park construction and update, and promotes the sustainable development of urban parks (29, 30, 31).

## 5. Conclusion

The subject of driving public health development by urban park system involves multiple cross disciplines, which complement and penetrate each other. This research comprehensively excavated the link elements among landscape science, psychology, public health management and other disciplines, and conducted in-depth research on the mechanism of urban park system driving public health from the two levels of “driving mechanism construction” and “public health planning thinking”:

In the future research on the relationship between urban parks and public health development, on the one hand, we should strengthen the quantitative research on the driving effect, analyze the causal relationship between urban parks and public health based on the correlation research, and fully consider the extent to which the urban park system promotes the healthy development of the public; On the other hand, take the application of artificial intelligence technology as the decision-making leader of urban park planning and design, use intelligent algorithms to conduct research in the preliminary feasibility study, better carry out the planning scheme layout and node refinement of urban park system, and further revise the interpretation framework of the urban park system driving public health mechanism proposed in this paper.

## Data availability statement

The original contributions presented in the study are included in the article/supplementary material, further inquiries can be directed to the corresponding author.

## Author contributions

CZ contributed the central idea, analyzed most of the data, wrote the initial draft of the paper, contributed to refining the ideas and carrying out additional analyses and writing—review and editing. YZ and QS contributed to writing—review and editing. All authors contributed to the article and approved the submitted version.

## Funding

This research was mainly supported by Anhui Provincial Key Research and Development Plan for Science and Technology Project “Key Technologies and Applications of Smart Tourism Services Based on Spatial Cloud Computing” (202104a07020002), The 2022 Anhui Provincial Scientific Research Preparation Plan Project (Scientific Research Project—Major Project; 2022AH040310), the Key Project of Scientific Research in Colleges and Universities of Anhui Provincial Department of Education (KJ2020A0835), the Ma’anshan City Land Inspection, Evaluation and Restoration Engineering Technology Research Center Open Fund (2020tdjc02), and Project of Engineering Technology Research Center for Sustainable Urban and Rural Human Settlements and Ecological Environment Planning, Wanjiang University of Technology (WGGCZX19001).

## Conflict of interest

The authors declare that the research was conducted in the absence of any commercial or financial relationships that could be construed as a potential conflict of interest.

## Publisher’s note

All claims expressed in this article are solely those of the authors and do not necessarily represent those of their affiliated organizations, or those of the publisher, the editors and the reviewers. Any product that may be evaluated in this article, or claim that may be made by its manufacturer, is not guaranteed or endorsed by the publisher.
